# A systematic approach to biomarker discovery; Preamble to "the iSBTc-FDA taskforce on immunotherapy biomarkers"

**DOI:** 10.1186/1479-5876-6-81

**Published:** 2008-12-23

**Authors:** Lisa H Butterfield, Mary L Disis, Bernard A Fox, Peter P Lee, Samir N Khleif, Magdalena Thurin, Giorgio Trinchieri, Ena Wang, Jon Wigginton, Damien Chaussabel, George Coukos, Madhav Dhodapkar, Leif Håkansson, Sylvia Janetzki, Thomas O Kleen, John M Kirkwood, Cristina Maccalli, Holden Maecker, Michele Maio, Anatoli Malyguine, Giuseppe Masucci, A Karolina Palucka, Douglas M Potter, Antoni Ribas, Licia Rivoltini, Dolores Schendel, Barbara Seliger, Senthamil Selvan, Craig L Slingluff, David F Stroncek, Howard Streicher, Xifeng Wu, Benjamin Zeskind, Yingdong Zhao, Mai-Britt Zocca, Heinz Zwierzina, Francesco M Marincola

**Affiliations:** 1 Department of Medicine, Division of Hematology Oncology, University of Pittsburgh Cancer Institute, Pittsburgh, Pennsylvania, 15213, USA; 2 Tumor Vaccine Group, Center for Translational Medicine in Women's Health, University of Washington, Seattle, Washington, 98195, USA; 3 Earle A Chiles Research Institute, Providence Portland Medical Center, Portland, Oregon, 97213, USA; 4 Department of Molecular Biology, OHSU Cancer Institute, Oregon Health and Science University, Portland, Oregon, 97213, USA; 5 Department of Medicine, Division of Hematology, Stanford University, Stanford, California, 94305, USA; 6 Cancer Vaccine Section, National Cancer Institute (NCI), National Institutes of Health (NIH), Bethesda, Maryland, 20892, USA; 7 Cancer Diagnosis Program, NCI, NIH, Rockville, Maryland, 20852, USA; 8 Cancer and Inflammation Program, NCI, NIH, Frederick, Maryland, 21702, USA; 9 Infectious Disease and Immunogenetics Section (IDIS), Department of Transfusion Medicine, Clinical Center and Center for Human Immunology, National Institutes of Health, Bethesda, MD, USA; 10 Bristol Myers-Squibb, Princeton, New Jersey, 08540, USA; 11 Baylor Institute for Immunology Research and Baylor Research Institute, Dallas, Texas, 75204, USA; 12 Center for Research on the Early Detection and Cure of Ovarian Cancer, University of Pennsylvania, Philadelphia 19104, USA; 13 Department of Hematology, Yale University, New Haven, Connecticut 06510, USA; 14 Division of Clinical Tumor Immunology, University of Lund, 581 85, Sweden; 15 ZellNet Consulting Inc. Fort Lee, New Jersey, 07024, USA; 16 Cellular Technology Limited, Shaker Heights, Ohio, 44122, USA; 17 Unit of Immuno-Biotherapy of Solid Tumors, Department of Molecular Oncology, San Raffaele Scientific Institute DIBIT, Milan, 20132, Italy; 18 Baylor Institute for Immunology Research, Dallas, 75204, Texas, USA; 19 Medical Oncology and Immunotherapy, Department. of Oncology, University Hospital of Siena, Istituto Toscano Tumori, Siena, Italy; 20 Cancer Bioimmunotherapy Unit, Department of Medical Oncology, Centro di Riferimento Oncologico, IRCCS, Aviano, 53100, Italy; 21 Laboratory of Cell Mediated Immunity, SAIC-Frederick, Inc., NCI-Frederick, Frederick, MD, 21702, USA; 22 Department of Oncology-Pathology, Karolinska Institute, Stockholm, 171 76, Sweden; 23 Biostatistics Department, Graduate School of Public Health, University of Pittsburgh, Pittsburgh, Pennsylvania, 15213, USA; 24 Department of Medicine, Jonsson Comprehensive Cancer Center, UCLA, Los Angeles, California, 90095, USA; 25 Unit of Immunotherapy of Human Tumors, IRCCS Foundation, Istituto Nazionale Tumori, Milan, 20100, Italy; 26 Institute of Molecular Immunology, and Clinical Cooperation Group "Immune Monitoring" Helmholtz Zentrum München, German Research Center for Environmental Health, Munich, 81377, Germany; 27 Institute of Medical Immunology, Martin-Luther University, Halle Wittenberg, Halle (Saale), 06112, Germany; 28 Hoag Cancer Center, Newport Beach, California, 92663, USA; 29 Department of Surgery, Division of Surgical Oncology, University of Virginia School of Medicine, Charlottesville, Virginia, 22908, USA; 30 Cell Therapy Section, Department of Transfusion Medicine, Clinical Center, NIH, Bethesda, Maryland, 20892, USA; 31 Cancer Therapy Evaluation Program, NCI, Bethesda, Maryland, 20852 USA; 32 Department of Epidemiology, University of Texas, MD Anderson Cancer Center, Houston, Texas, 77030, USA; 33 Immuneering Corporation, Boston, Massachusetts, 02215, USA; 34 Biometrics Research Branch, NCI, NIH, Bethesda, Maryland, 20852, USA; 35 DanDritt Biotech A/S, Copenhagen, 2100, Denmark; 36 Department of Internal Medicine, Innsbruck Medical University, Innsbruck, 6020, Austria

## Abstract

The International Society for the Biological Therapy of Cancer (iSBTc) has initiated in collaboration with the United States Food and Drug Administration (FDA) a programmatic look at innovative avenues for the identification of relevant parameters to assist clinical and basic scientists who study the natural course of host/tumor interactions or their response to immune manipulation. The task force has two primary goals: 1) identify best practices of standardized and validated immune monitoring procedures and assays to promote inter-trial comparisons and 2) develop strategies for the identification of novel biomarkers that may enhance our understating of principles governing human cancer immune biology and, consequently, implement their clinical application. Two working groups were created that will report the developed best practices at an NCI/FDA/iSBTc sponsored workshop tied to the annual meeting of the iSBTc to be held in Washington DC in the Fall of 2009. This foreword provides an overview of the task force and invites feedback from readers that might be incorporated in the discussions and in the final document.

## Background

Assumptions about correlation between immunological end-points and clinical outcomes of immunotherapy or anti-cancer vaccine therapy are not supported by current monitoring strategies; standard immunological assays may inform about immunological outcomes but cannot yet predict the efficacy of treatment [1].

The failure of past clinical investigations to identify measurable, reliable biomarkers predictive of treatment efficacy may be explained two ways:

A. The current understanding of the immune biology of tumor/host interactions and the immunological requirements for the induction of immune-mediated, tissue-specific destruction is insufficient. Thus, novel hypothesis-generating strategies should be considered.

B. The power of immunotherapy clinical studies is often not sufficient to provide robust statistical information because of their small size and because the immune assays are not sufficiently standardized or broad to allow inter-trial, inter-institutional comparisons to enhance statistical power.

To address the first point, a working group (*Novel Assays for Immunotherapy Clinical Trials*) has been organized under the leadership of Peter Lee and Francesco Marincola aimed at the identification of experimental, bioinformatics and clinical strategies to increase the yield of information relevant to the mechanism of immune-mediated, tissue-specific rejection to develop clinically useful markers and assays.

To address the second point, another working group (*Biomarker Validation and Application*) has been organized under the leadership of Lisa Butterfield, Nora Disis and Karolina Palucka to evaluate current approaches to the validation of known immune response biomarkers and the standardization of the respective assays to enhance the likelihood of obtaining informative returns from ongoing immunotherapy protocols at different institutions. This working group will focus primarily on the standardization and corroboration of commonly utilized assays for measurement of host-tumor interaction and immune response to therapeutic intervention; in addition, it will develop best practices for the standardization and corroboration of novel assays.

### Working group on novel assays for immunotherapy clinical trials

Co-Chairs: Peter P Lee, MD – Stanford University

Francesco M Marincola MD – Clinical Center, NIH

### Goals

This working group goal consists of testing novel, cutting-edge strategies suitable for high-throughput screening of clinical samples for the identification, selection and validation of biomarkers relevant to disease outcome and/or to serve as surrogate equivalents to clinical outcome. In particular, the working group will focus on:

A. Predictors of immune responsiveness are defined as a set of biomarkers that could predict at the time of patient's enrollment her/his responsiveness to treatment [2,3]. This type of markers will be particularly important in immunotherapies since standard response criteria (RECIST and WHO) to define tumor response and disease progression (tumor shrinkage) might not adequately capture the clinical benefit. In immunotherapy trials, some patients demonstrate long-term survival benefit from treatment but delayed responses and show continued tumor growth initially [4]. By standard criteria, such patients would be classified as having progressive disease and taken off study.

B. Markers predicting risk of toxicity are defined as biomarkers that could predict at the time of patient's enrollment her/his likelihood to suffer major toxicity from a specific therapy.

C. Mechanistic biomarkers are defined as those that may explain or validate the mechanism(s) of action of a given treatment in humans; such biomarkers will be more likely identified by paired comparison of pre- and post-treatment samples[5]. Critical to the design of studies aimed at the identification of mechanistic biomarkers will be the inclusion of relevant control samples to allow the differentiation between treatment related effects from the effects on tissues of serial biopsies that induce wound repair associated genes and proteins [6].

D. Prognostic markers predicting survival/clinical benefit could predict overall outcome independent of clinical responsiveness based on standard response criteria [7,8].

E. Surrogate (end-point) biomarkers are defined as those biomarkers that could provide information about the likelihood of clinical benefit/survival at earlier stages compared to prolonged disease-free or overall survival analysis.

The goals of this working group are especially challenging since there are multiple categories of immunotherapies having their own complexities often representing multi-component systems such as vaccines. Nevertheless, there is a need for biomarkers to determine the effect of the drug on the tumor as well as assessment of the host immune response. Thus, the goals are broader and less restrictive than those of the *working group on Biomarker Validation and Application *because specific challenges to the identification and validation of biomarkers using novel and rapidly evolving approaches have been less clearly characterized. Consequently, the establishment of sub-committees addressing specific issues is planned at a later time either before or after the 2009 workshop when defined scientific or practical hurdles will be prioritized and framed into specific questions. Furthermore, the selection and implementation of different sub-committees will follow an adhocracy model according to evolving and progressively recognized needs [9].

### Basic considerations

Success will only be achieved by boldly following new strategies likely to provide informative data independent of other practical or financial considerations. In other words, a study should be primarily designed following rigorous and stringent criteria that allow the achievement of its scientific goals. As the design proceeds to the implementation phase, other considerations should obviously be taken into consideration and negotiated carefully, optimizing the balance between them and the likelihood to obtain the originally desired outcomes. A good example is the implementation of serial sampling for mechanistic studies [10]; such strategies have been discussed for a long time but rarely applied due to a hesitant attitude on the side of clinicians. On the other hand, examples of the applicability of such strategies in institutionally approved protocols is emerging because of the enormous scientific return that can be obtained from these kinds of studies [5,11,12]. Therefore, the basic belief in relation to the purposes of this working group is that a clinical study should be entertained only if likely to provide significant enhancement of the science of immunotherapy; in other words, a poorly designed clinical study is worse than no study at all. Furthermore, identification of novel and relevant biomarkers should be sought by prospectively designing clinical studies with that purpose rather than piggybacking ongoing studies.

Marker discovery/development for immunotherapy is especially challenging since humans are:

i. Polymorphic

ii. Tumors are heterogeneous

iii. Environmental conditions variably affect tumor development/progression

None of these factors are controllable. Therefore, future studies should confront the challenges of clinical investigation by accruing materials that could comprise the genetic background of patients, the heterogeneity of their cancers and other indeterminate factors that may contribute to patients' and cancer cell phenotypes. This goal can likely be achieved through a non-linear mathematical approach based on pattern recognition [13-15]. The leading hypothesis is that, within a heterogeneous system, commonalities observed during the occurrence of a particular phenomenology (i.e. response to therapy) are most likely to be relevant and/or causative [16]. Thus, the general strategy will be to obtain:

i. Samples to address the genetic background of the patients (germ line DNA, i.e. peripheral blood mononuclear cells, PBMCs)

ii. Samples to address the altering phenotypes of immune cells in relation to the natural history of disease and/or treatment (i.e. pre, during, and post-treatment PBMCs, sera or plasma at the same time points, pre-treatment and/or serial biopsies) that could provide insights about the identification of biomarkers predictive of responsiveness or toxicity.

iii. Samples that may provide mechanistic insights about the relationship between tumor biology and treatment (i.e. tumor biopsies, sentinel node biopsy etc).

Appropriate sample collection should be considered the independent variable while the technologies applied for their analysis may rapidly evolve and will have to adjust; experts in various fields of genomics, functional genomics, and proteomics will provide useful insights. In addition, recent interest has risen toward the characterization of cellular products, tissue or genetically engineered products for adoptive transfer by high throughput technologies including transcriptional profiling at the messenger RNA [17] and microRNA [18] level.

It should be emphasized that there is no priority scale about which of the three lines of investigation is most important; indeed, only the combination of them can provide a global view of the pathological process. Furthermore, questions regarding the type of material to be utilized (i.e., DNA, RNA or proteins) underline some naiveté in the way clinical investigations may be approached. In an oversimplified view, humans, as multi-cellular organisms, are structured according to a hierarchy of genetic interactions that go from genomic DNA, to transcription into RNA and translation into functional units (proteins in different functional statuses) that may or may not differ among cells within a tissue or from different tissues. The study of each layer within this hierarchy provides distinct information: DNA analysis provides information about relatively stable characteristic of cells and tissues that may explain variations among individual patients, or aberrances between normal and abnormal tissues; messenger RNA informs mostly about the reaction of cells to environmental conditions; we compare transcriptional analysis to the electroencephalographic responses to stimulation which inform about the reaction to stimulus; thus, while mRNA provides information about the "brain response" of a cell (spikes in response to light), protein analysis (including functional assays descriptive of protein activation [19] and/or expression by immune cell subsets [20]) provides information about what a cell is doing as the hand covers the eyes when the light is too strong. Since each component provides different types of information and one kind cannot be assumed from the other, clinical research should study humans by evaluating all components simultaneously at moments relevant to the natural history of a disease or its response to therapy. Of importance is the realization that protein analysis confronts particular challenges when studying immunologically relevant soluble factors that are generally present in low concentrations (though biologically significant) in body fluids like serum or plasma [21] and potentially exist as isoforms with different functional implications [22].

Advances in metabolic imaging based on positron emitting tomography (PET) and in sensitive protein assays based on nanotechnology platforms provide the promise of non-invasive and minimally-invasive immune monitoring. The use of PET-based probes preferentially taken up by activated T cells enables non-invasive imaging of immune responses *in vivo *without perturbing the biological process with blood cell or tissue sampling [23,24]. In addition, the increased knowledge of the proteins secreted during immune activation and tumor cell killing (secretome) can be detected in small volume serum samples (ideally from a finger-prick) when analyzed by high throughput nanotechnology-based assays [25,26]. These new technologies applied to immune monitoring would enable the sequential and repetitive analysis of an effective immune response. Ideally, the novel assay technologies will need to first be compared to more standard approaches to define their analytical bias, leading to adequate correlation with biological processes and clinical outcomes. Furthermore, circulating RNA profiling measures predominantly transcriptional activation of circulating cells, while protein profiling measures abundance of proteins produced by several tissues.

#### General strategy

Experience from non-linear, pattern-recognizing approaches such as whole genome analysis or functional genomics suggest that the best and most efficient statistical strategy for biomarker identification/validation is a two (three) step process that includes:

##### i. A discovery/training step

This step may require a relatively limited number of samples to be tested extensively to identify putative informative pathways or genetic traits using costly, high-throughput and comprehensive strategies.

##### ii. A training/validation step

This bears the same characteristics of the training set with two exceptions: a) should be performed by an independent group; b) could be better powered because the study can be designed with a priori knowledge of experimental variance.

##### iii. A validation step

The validation set follows to validate the previously identified pathway or genetic trait using less costly and more focused analyses on larger patient populations. Thus, the validation step bears the same characteristics of the training/discovery set but it should be performed in a large independent specimen cohort sufficient to provide the results to support the clinical use of the marker (prognostic response, toxicity, etc.). It should include a clear statistical design to assure the marker correlation with the clinical parameter of interest

Key to successful implementation of this strategy is the decision to move from the "discovery phase" (training set) to the "validation phase". Arguably, in the past the scientific community has been too eager to move from the first to the second without substantial evidence that the first phase had been truly completed. It could be argued that a second "training/validation" set should be added to independently test the reproducibility of the results in a small cohort; several strategies may be adopted including a paired performance of identical studies at two different institutions blinded about each others results. Bioinformatic and statistical support are critical in defining the most effective and least time-consuming strategies and we advocate that a biostatistician/computational biologist should play a significant role in the committee. Moreover, the separation between training and validation phases is critical because sample collection, storage and utilization may significantly vary; less material may be required during the validation step when narrower questions are approached. However, while some features of sample collection may change, experimental consistency will not be negotiable. The three step strategy may be able to provide the highest yield of information during the transition from a high cost per patient during the exploratory phase to a less costly per patient but highly powered validation phase. Bioinformatics and statistical support are critical in defining the most effective and least time-consuming strategies and we advocate that a biostatistician, computational biologist should play a significant role in the working group starting from the clinical study design.

#### Strategy for sample collection

A working hypothesis of the working group is that the biggest obstacle to the identification of useful biomarkers is the difficulty in obtaining relevant material to study, while the potential of current technologies is proportionally limitless. Due to practical, ethical and financial rationalizations, samples are rarely collected with a methodology that allows broad testing opportunities and at a time or anatomical site relevant to the question asked. The working group will address each of these questions by including a bioethicist, members of regulatory agencies and a statistician together with the clinical and research input provided by other members and, potentially, patients' advocacy groups. The contention is that 1) excessive and unnecessary regulatory burdens ultimately result in a disservice to present and future patients, 2) studies limited for financial reasons are likely to be more wasteful than well-designed costly studies because they will eventually need to be repeated; 3) the application of training/validation strategies may significantly reduce costs without compromising the scientific yield of well-designed studies. Strategies for sample collection include the following:

##### i. Time of collection

The time of collection critically impacts functional studies. Obviously, it is less important when analyzing the genetic background of individuals since germ line DNA does not change throughout the natural history of the disease. However, functional studies involving the utilization of messenger RNA or protein from samples before and during treatment are highly affected by the rapid kinetics of the immune response and the evolving nature of cancer cell phenotypes.

##### ii. Method of collection

Clinical samples are often difficult to obtain, impractical and require invasive technology. Although these are important considerations, none should compromise the collection of informative material. Non-invasive technologies have been developed, validated and optimized during the last decade to improve the feasibility of high-throughput studies in clinical settings [10]. Furthermore, use of anti-coagulants and/or other preservatives may have significant impact on measurements [27].

##### iii. Method of preservation

Strategies can be implemented to preserve materials prospectively in selected cohorts of patients (training set strategy) to improve the quality of the specimens; rapid freezing methods, use of anti-proteases or anti-RNAase, aliquoting of material to avoid serial freeze-and-thaw cycles. These precautions will increase significantly the likelihood of obtaining informative results by reducing variance.

##### iv. Type of sample

DNA, RNA and protein material should be obtained whenever possible. Germ-line DNA is important for testing genetic predisposition/influence on treatment outcome. However, genetic testing often requires a large number of cases due to the functional redundancy of human genes and the co-segregation of genetic traits according to geo-ethnical origin independent of specific phenomenologies. Expertise from immunogeneticists will be important. Transcriptional analysis has matured during the last decade and expertise in RNA handling and amplification will be present in the working group. A protein biochemist will be included that could provide expertise about the sample handling and research approaches appropriate for immunological studies (i.e. low concentration of cytokines and chemokines below the sensitivity of present discovery-driven proteomic approaches).

##### v. Number of samples

Individual protocols will require a different number of samples to achieve the same statistical power according to the variance expected in the study population and its responsiveness to therapy and/or susceptibility to toxic side effects (i.e. the expected frequency of responders to a given treatment will dictate the size of training and prediction set). Moreover, definition in mathematical terms of biological equivalence *vs *diversity of cellular and biological products will be discussed (i.e. what parameter defines equality or difference of dendritic cell processing following "identical" procedures).

##### vi. Methods of analysis

Concerns often focus on methods for sample collection and storage and validation and cross-validation on novel technologies. We believe that the significance of these concerns is overrated, particularly in the case of hypothesis-generating studies where the main goal is to screen clinical material for the identification of novel ideas to be validated later on by other techniques. This opinion is based on evidence that results obtained by various groups collimate conceptually with results obtained by others using different platforms and samples and with common sense biological knowledge [28-32]. As human biology is an independent variable, different platforms applied to its study should provide concordant results as the essence of life is not changed by the spectacles through which we observe it, though our perceptions might vary from jolly to gloomy in accordance with the pink or dark lenses that we wear. This is critical in clinical research: by far, the key concern should be timing, site and method of sample accrual while rapidly evolving technologies will have to adapt to what is available and worth studying. Although counterintuitive, the methods applied for the study are less critical than the quality of the material accrued. Experience with various functional genomics platforms suggest that results are quite comparable as long as the same material is tested but most discrepancies occur when studies performed at different institutions or on samples received from different institutions are compared. The potentials of modern technology are proportionately limitless and flexible; bioinformatics tools can robustly evaluate concordance of results, identify consistent and random biases and sieve reliable data. As technology rapidly evolves, tools can be adapted to compare platforms and provide biologically consistent results. Thus, although the quality of the material will remain a primary focus of the working group, the need for platform standardization or, at least comparability of results to facilitate inter-trial, inter-institutional comparisons will be a focus of discussion. Furthermore, the definition used for the collection of clinical information or metadata derived from the bedside vary widely and are likely to make the task of consolidating clinical trials results even more daunting.

##### vii. Standardization, Centralization, Validation

Although the principles of standardization and validation of assays are the primary purpose of the working group on "*Biomarker Validation and Application*", sound strategies should be applied to address the imminent needs of the present working group evaluating novel technologies in uncharted territories; it is our opinion that assay standardization is most important in the early phases of biomarker discovery when limited sample size of different protocols can be counterbalanced by the accumulation of comparable results from different studies/institutions. Thus, the following concepts will be considered:

##### i. Standardization

It is generally difficult to enforce standardization of methods when novel technologies are approached due to the unsolved biases among individual investigators about the pros and cons of emerging technologies. Thus, standardization could be enforced by proposing standardization of sample collection (comparable material) and cross validation of the samples among different institutions to assure similar results independent of platform used.

##### ii. Sample exchange

The comparability of results could be compared by exchange of training samples among trials/institutions. This may obviate biased selection of platforms based on limited knowledge about their pros and cons.

##### iii. Centralization

A super core facility could support the analysis of samples from different but comparable trials as, for instance, the novel Center for Human Immunology which is part of an inter-NIH initiative with pre-dominant intra-mural scopes but open to extra-mural interactions.

##### iv. Validation

it is important to distinguish between these two concepts: 1) assay validation; 2) biomarker validation

1. **Assay validation**: is not the purpose of this working group; validation of assay deemed useful by this working group will be performed by the sister working group after discussion of its potential benefits.

2. **Biomarker validation**: potential discovery of a new robust candidate as a biomarker will need to be validated by a validation set as described above: this is part of the goals of the working group; arguably, a robust biomarker should be useful independent of the test applied. In general, concordant results about the validity of a biomarker by different platforms should provide stronger confidence about its clinical relevance. Hence, this working group will not focus particular attention on assay validation but rather on biomarker validation.

#### Data exchange

Data collection and data exchange is becoming extremely burdensome: a whole genome SNP array from Affymetrix requires approximately 1 Gbyte of memory. Data exchange requires compatible databases and similar languages which are not readily available. Thus, informatics distances are large in spite of the disruption of geographical distances through the *World Wide Web*. Centralization of information may represent a solution as exemplified by the Center of Information Technology at NCI that standardizes and collects all high-density data for the intra-mural program. Similarly, data analysis could be centralized as several inter-institutional cooperative groups are already doing for low density data handling. Large *bioinformatics wastelands *could be avoided if data could be effectively mined by various groups interested in similar problems; however, in our experience this seldom occurs due to the complexity of exchanging basic information about the strategies in which data bases were prepared particularly considering the little incentive due to little funding available for re-analysis and unclear publication opportunities.

### Working group on biomarker validation and application

Co-Chairs: Lisa H. Butterfield, PhD – University of Pittsburgh

Nora Disis, MD – University of Washington

A. Karolina Palucka, MD, PhD – Baylor Institute for Immunology Research

### Desired outcomes

This working group has clearly defined goals that can be summarized as follows:

1) Identification of recommended SOPs for blood, serum/plasma and PBMC transportation, processing, cryopreservation and thawing. Many of these have been previously tested, standardized and published [33-35]. Specific protocols and SOPs should be posted on the web and broadly available for use and citation. In addition, sample collection and storage should take into account new assays. Similar considerations should be taken into account when collecting sera or plasma during the conduct of clinical trials [36].

2) The identification of specific standardized and validated immunological assays for both potency of products and testing of immunologic biomarkers which incorporate intra-assay and inter-assay reference standards for comparison between laboratories and potentially between clinical trials, as well as standardization of assay data reporting. Again, there have been many reports published in these areas [37], and this group proposes to review the state of the art, including recent undertakings of related international societies, and present a consensus. Our goals are to identify a few assays which are minimally required in a trial to identify successfully vaccinated patients and patients who would respond to specific immunotherapy (and to allow for potential inter-trial comparisons). Also, the activity of this group will focus on criteria for assessment of analytical range and sensitivity, accuracy, precision and reproducibility for assay validation. The group will also identify the most commonly used assay controls and reagents which might be recommended and made available for common use. Recommended cellular product potency assays should be tested now, in Phase I/II trials, in preparation for use in any Phase III trials.

Lastly, 3) the integration of standardized and/or validated assays (with recommended data reporting parameters) into new clinical trial design and outcome structure will be recommended.

### Critical Issue for discussion

How to take best advantage of the work in the infectious disease and immune tolerance fields where much standardization has already been worked through and implemented?

### Charges

*1. *Identification of validated SOPs for blood handling and transportation, processing, cryopreservation and thawing, with new assays in mind.

*2. *Development of guidelines for pre-analytical standardization, requirements for assay validation and results reporting that meet CLIA requirements.

*3. *Development of scientifically sound and statistically significant definitions of immune response based on immune monitoring assays. This would require defining the performance specifications within the reportable range of the assay, as described [38]. Assays should specify whether they are quantitative or semi-quantitative, the scoring system and threshold values that differentiate between responders and non responders must be specified.

*4. *Source for standard cell lines (T2, K562/A2.1, etc.) and culture SOPs.

*5. *Identification of potency assays for cellular products for development and testing in current immunotherapy trials: a) cellular vaccine phenotypes (DC, other APC, CTL/TIL, NK, NK/T), b) cytokine/chemokine production, c) antigen uptake/presentation and d) functional assessment [39].

*6. *Develop specific guidelines for detection of T cell frequencies: IFN-γ ELISPOT [40] and for "other cytokine" ELISPOTs, intracellular cytokine staining, cytotoxicity assays, proliferation, (focus on non radioactive and multi-parameter), specific antigen ELISA/Luminex and MHC class I tetramer flow cytometry. For most routine assays, a simple statement of general parameters with citations.

*7. *Develop strategies for standardization and validation of monitoring non-HLA-A2.1 patients, particularly the use of long peptides, peptide libraries and full-length antigens.

*8. *Identification of a few core assays which are minimally required in a trial to identify successfully vaccinated patients and/or patients who respond to a specific immunotherapy. Particularly, the least costly assay which is standardized and/or validated, with freely available reference standards which can be used in each assay run. This should include specific recommendations for assay parameters, coefficient of variation (CV) and data analysis to report in publications. This should also include defining the analytical variation of the assay as well as determining the biological fluctuations of antigen-specific T cells in humans over time in the absence of an intervention [41].

*9. *Development of assay reference standards that meet CLIA requirements. Recommend optimal sources of critical reagents.

*10. *Identification of scientific areas in which assays should be developed, including apoptosis, myeloid-derived suppressor cells, tumor microenvironment assessment, discussion of issues inherent to antigen-specific DTH testing, and T regulatory cells assessment. This should be based on a systematic approach of method selection, evaluation, development and implementation (specific recommendations available on the web, [42].

There are increasingly frequent reports of statistically significant correlations between measures of anti-tumor immunity and clinical outcome. Greater standardization is required to strengthen these associations and provide more mechanistic insights to inform future trial design. In addition, utilization of CLIA-certified and inspected central laboratories allows for standardization of most aspects of assay conduct and also for cost effective assay development and validation.

### Expected milestones for both working groups

• The 2009 iSBTc Workshop preceding the 2009 Annual Meeting [1].

• Preparation of a document with input from all participants at the end of the task force to be published after the 2009 Workshop (as done in previous occasions [43,44]).

• Provision of links to recommended SOPs and the resultant document on the iSBTc web site with links to the web sites of participating societies and organizations.

### Expected outcomes of the taskforce

• Potential collaborations among different laboratories, institutions, companies and international societies which are also focused on similar efforts of standardization and harmonization of goals.

• Development of cooperative groups for the study design, identification and sharing of resources, centralization of analyses in core laboratories, establishment of ad hoc tissue and data banks and development of easy to access data repositories.

## Competing interests

The authors declare that they have no competing interests.

## Authors' contributions

LHB, MLD, BAF, PPL, SNK, MT, JW and FMM are part of the Biomarkers Task Force Steering Committee and prepared the original draft of this document; the other authors (GT, EW, DC, GC, MD, LH, SJ, TK, JK, CM, HM, MM, AM, GM, AKP, DMP, AR, LR, DS, BS, SS, GLS Jr, DFS, HS, XX, BZ, YZ, M-B Z, HZ) contributed to the preparation of the final draft with comments and additions.

## References

[B1] iSBTc (2008). iSBTC/FDA Immunotherapy Biomarker Taskforce. http://www.isbtc.org/news/enews.php.

[B2] Atkins MB, Regan M, McDermott D, Mier J, Stanbridge E, Youmans A, Febbo P, Upton M, Lechpammer M, Signoretti S (2005). Carbonic anhydrase IX expression predicts outcome in interleukin-2 therapy of renal cancer. Clin Cancer Res.

[B3] Sabatino M, Kim-Schulze S, Panelli MC, Stroncek DF, Wang E, Tabak B, Kim DW, De Raffaele G, Pos Z, Marincola FM, Kaufman H (2008). Serum vascular endothelial growth factor (VEGF) and fibronectin predict clinical response to high-dose interleukin-2 (IL-2) therapy. J Clin Oncol.

[B4] Wolchok JD, Chapman PB (2003). How can we tell when cancer vaccines vaccinate?. J Clin Oncol.

[B5] Panelli MC, Stashower M, Slade HB, Smith K, Norwood C, Abati A, Fetsch P, Filie A, Walters SA, Astry C, Aricó E, Zhao Y, Selleri S, Wang E, Marincola FM (2006). Sequential gene profiling of basal cell carcinomas treated with Imiquimod in a placebo-controlled study defines the requirements for tissue rejection. Genome Biol.

[B6] Deonarine K, Panelli MC, Stashower ME, Jin P, Smith K, Slade HB, Norwood C, Wang E, Marincola FM, Stroncek DF (2007). Gene expression profiling of cutaneous wound healing. J Transl Med.

[B7] Zhang L, Conejo-Garcia JR, Katsaros D, Gimotty PA, Massobrio M, Regnani G, Makrigiannakis A, Gray H, Schlienger K, Liebman MN, Rubin SC, Coukos G (2003). Intratumoral T cells, recurrence, and survival in epithelial ovarian cancer. N Engl J Med.

[B8] Galon J, Costes A, Sanchez-Cabo F, Kirilovsky A, Mlecnik B, Lagorce-Pages C, Tosolini M, Camus M, Berger A, Wind P, Zinzindohoué F, Bruneval P, Cugnenc PH, Trajanoski Z, Fridman WH, Pagès F (2006). Type, density, and location of immune cells within human colorectal tumors predict clinical outcome. Science.

[B9] Mintzberg H (1981). Organizational design, fashion or fit?. Harvard Business Rev.

[B10] Wang E, Marincola FM (2000). A natural history of melanoma: serial gene expression analysis. Immunol Today.

[B11] Wang E, Miller LD, Ohnmacht GA, Mocellin S, Petersen D, Zhao Y, Simon R, Powell JI, Asaki E, Alexander HR, Duray PH, Herlyn M, Restifo NP, Liu ET, Rosenberg SA, Marincola FM (2002). Prospective molecular profiling of subcutaneous melanoma metastases suggests classifiers of immune responsiveness. Cancer Res.

[B12] Panelli MC, Wang E, Phan G, Puhlman M, Miller L, Ohnmacht GA, Klein HG, Marincola FM (2002). Gene-expression profiling of the response of peripheral blood mononuclear cells and melanoma metastases to systemic IL-2 administration. Genome Biol.

[B13] Chaussabel D, Quinn C, Shen J, Patel P, Glaser C, Baldwin N, Stichweh D, Blankenship D, Li L, Munagala I, Bennett L, Allantaz F, Mejias A, Ardura M, Kaizer E, Monnet L, Allman W, Randall H, Johnson D, Lanier A, Punaro M, Wittkowski KM, White P, Fay J, Klintmalm G, Ramilo O, Palucka AK, Banchereau J, Pascual V (2008). A modular framework for biomarker and knowledge discovery from blood transcriptional profiling studies: application to systemic lupus erythemathosus. Immunity.

[B14] Wang E, Marincola FM (2008). Bottom up: a modular view of immunology. Immunity.

[B15] Gabriele L, Moretti F, Pierotti MA, Marincola FM, Foa R, Belardelli F (2006). The use of microarray technologies in clinical oncology. J Transl Med.

[B16] Wang E, Worschech A, Marincola FM (2008). The immunologic constant of rejection. Trends Immunol.

[B17] Stroncek DF, Jin P, Wang E, Jett B (2007). Potency analysis of cellular therapies: the emerging role of molecular assays. J Transl Med.

[B18] Jin P, Wang E, Ren J, Childs R, Shin JW, Khuu H, Marincola FM, Stroncek DF (2008). Differentiation of two types of mobilized peripheral blood stem cells by microRNA and cDNA expression analysis. J Transl Med.

[B19] Marks KM, Nolan GP (2006). Chemical labeling strategies for cell biology. Nat Methods.

[B20] Beasley JR, McCoy PM, Walker TL, Dunn DA (2004). Miniaturized, ultra-high throughput screening of tyrosine kinases using homogeneous, competitive fluorescence immunoassays. Assay Drug Dev Technol.

[B21] Rossi L, Martin B, Hortin G, White RLJr, Foster M, Stroncek D, Wang E, Marincola FM, Panelli MC (2006). Inflammatory protein profile during systemic high dose interleukin-2 administration. Proteomics.

[B22] Rossi L, Moharram R, Martin BM, White RL, Panelli MC (2006). Detection of human MCP-4/CCL13 isoforms by SELDI immunoaffinity capture. J Transl Med.

[B23] Radu CG, Shu CJ, Nair-Gill E, Shelly SM, Barrio JR, Satyamurthy N, Phelps ME, Witte ON (2008). Molecular imaging of lymphoid organs and immune activation by positron emission tomography with a new [18F]-labeled 2'-deoxycytidine analog. Nat Med.

[B24] Tumeh PC, Radu CG, Ribas A (2008). PET imaging of cancer immunotherapy. J Nucl Med.

[B25] Bailey RC, Kwong GA, Radu CG, Witte ON, Heath JR (2007). DNA-encoded antibody libraries: a unified platform for multiplexed cell sorting and detection of genes and proteins. J Am Chem Soc.

[B26] Fan R, Vermesh O, Srivastava A, Yen BK, Qin L, Ahmad H, kwong GA, Liu CC, Gould J, Hood L, Heath JR (2008). Integrated barcode chips for rapid, multiplexed analysis of proteins in microliter quantities of blood. Nat Biotechnol.

[B27] Ayache S, Panelli M, Marincola FM, Stroncek DF (2006). Effects of storage time and exogenous protease inhibitors on plasma protein levels. Am J Clin Pathol.

[B28] Jin P, Zhao Y, Ngalame Y, Panelli MC, Nagorsen D, Monsurro' V, Smith K, Hu N, Su H, Taylor PR, Marincola FM, Wang E (2004). Selection and validation of endogenous reference genes using a high throughput approach. BMC Genomics.

[B29] Wang E, Panelli MC, Zavaglia K, Mandruzzato S, Hu N, Taylor PR, Seliger B, Zanovello P, Freedman RS, Marincola FM (2004). Melanoma-restricted genes. J Transl Med.

[B30] Jin P, Wang E, Provenzano M, Deola S, Selleri S, Jiaqiang R, Voiculescu S, Stroncek D, Panelli MC, Marincola FM (2006). Molecular signatures induced by interleukin-2 on peripheral blood mononuclear cells and T cell subsets. J Transl Med.

[B31] Basil CF, Zhao Y, Zavaglia K, Jin P, Panelli MC, Voiculescu S, Mandruzzato S, Lee HM, Seliger B, Freedman RS, Taylor PR, Hu N, Zanovello P, Marincola FM, Wang E (2006). Common cancer biomarkers. Cancer Res.

[B32] Fang W, Li X, Jiang Q, Liu Z, Yang H, Wang S, Xie S, Liu Q, Liu T, Huang J, Xie W, Li Z, Zhao Y, Wang E, Marincola FM, Yao K (2008). Transcriptional patterns, biomarkers and pathways characterizing nasopharyngeal carcinoma of Southern China. J Transl Med.

[B33] Maecker HT, Moon J, Bhatia S, Ghanekar SA, Maino VC, Payne JK, Kuus-Reichel K, Chang JC, Summers A, Clay TM, Morse MA, Lyerly HK, DeLaRosa C, Ankerst DP, Disis ML (2005). Impact of cryopreservation on tetramer, cytokine flow cytometry, and ELISPOT. BMC Immunol.

[B34] Disis ML, dela Rosa C, Goodell V, Kuan LY, Chang JC, Kuus-Reichel K, Clay TM, Kim Lyerly H, Bhatia S, Ghanekar SA, Maino VC, Maecker HT (2006). Maximizing the retention of antigen specific lymphocyte function after cryopreservation. J Immunol Methods.

[B35] Ghanekar SA, Bhatia S, Ruitenberg JJ, dela RC, Disis ML, Maino VC, Maecker HT, Waters CA (2007). Phenotype and in vitro function of mature MDDC generated from cryopreserved PBMC of cancer patients are equivalent to those from healthy donors. J Immune Based Ther Vaccines.

[B36] Ayache S, Panelli MC, Byrne KM, Slezak S, Leitman SF, Marincola FM, Stroncek DF (2006). Comparison of proteomic profiles of serum, plasma, and modified media supplements used for cell culture and expansion. J Transl Med.

[B37] Maecker HT, Hassler J, Payne JK, Summers A, Comatas K, Ghanayem M, Morse MA, Clay TM, Lyerly HK, Bhatia S, Ghanekar SA, Maino VC, Delarosa C, Disis ML (2008). Precision and linearity targets for validation of an IFNgamma ELISPOT, cytokine flow cytometry, and tetramer assay using CMV peptides. BMC Immunol.

[B38] Fraser CG (2001). Biological Variation: from Principles to Practice.

[B39] Butterfield LH, Gooding W, Whiteside TL (2008). Development of a potency assay for human dendritic cells: IL-12p70 production. J Immunother.

[B40] Janetzki S, Panageas KS, Ben-Porat L, Boyer J, Britten CM, Clay TM, Kalos M, Maecker HT, Romero P, Yuan J, Kast WM, Hoos A, Elispot Proficiency Panel of the CVC Immune Assay Working Group (2008). Results and harmonization guidelines from two large-scale international Elispot proficiency panels conducted by the Cancer Vaccine Consortium (CVC/SVI). Cancer Immunol Immunother.

[B41] Comin-Anduix B, Gualberto A, Glaspy JA, Seja E, Ontiveros M, Reardon DL, Renteria R, Englahner B, Economou JS, Gomez-Navarro J, Ribas A (2006). Definition of an immunologic response using the major histocompatibility complex tetramer and enzyme-linked immunospot assays. Clin Cancer Res.

[B42] (2008). Westgard QC: Tools, Technology and Training for Healthcare Laboratories. http://www.westgard.com.

[B43] Keilholz U, Weber J, Finke J, Gabrilovich D, Kast WM, Disis N, Kirkwood JM, Scheibenbogen C, Schlom J, Maino VC, Lyerly HK, Lee PP, Storkus W, Marincola F, Worobec A, Atkins MB (2002). Immunologic monitoring of cancer vaccine therapy: results of a Workshop sponsored by the Society of Biological Therapy. J Immunother.

[B44] Lotze MT, Wang E, Marincola FM, Hanna N, Bugelski PJ, Burns CA, Coukos G, Damle N, Godfrey TE, Howell WM, Panelli MC, Perricone MA, Petricoin EF, Sauter G, Scheibenbogen C, Shivers SC, Taylor DL, Weinstein JN, Whiteside TL (2005). Workshop on cancer biometrics: identifying biomarkers and surrogates of cancer in patients. J Immunother.

